# What will the ambitions of primary healthcare be in the 21^st^ century?

**DOI:** 10.11604/pamj.2022.43.87.35235

**Published:** 2022-10-19

**Authors:** Nasreddine Aissaoui, Lamia Hamaizia, Said Khalfa Brika, Talaat Rashad Abd Elfatah Shama

**Affiliations:** 1 Department of Finance Sciences, Faculty of Economics, Business and Management Sciences, Oum El Bouaghi University, Oum El Bouaghi, Algeria,; 2 Department of Management Sciences, Faculty of Economics, Business and Management Sciences, Oum El Bouaghi University, Oum El Bouaghi, Algeria,; 3 Departement of Administrative Sciences, Applied College, University of Bisha, Bisha, Saudi Arabia,; 4 Department of Business Administrations, College of Business, University of Bisha, Bisha, Saudi Arabia

**Keywords:** Primary healthcare, reform of primary healthcare system, modernity of primary healthcare

## Abstract

This article attempts to analyze the problem of the modernity of primary healthcare, as well as the reforms to be implemented in a new context characterized by COVID-19. This article offers another vision to follow, to build a modern primary healthcare system. It is a descriptive and analytical study, which addresses the failure of the health system in general, as it proposes the reforms necessary to provide equitable, efficient and modern primary healthcare. The results clearly show that we can no longer act unilaterally; multi-sector efforts at the national level should be encouraged: we must act, in a preventive way, on the causes of the disease; international agreements on the environment, prices of basic foods and medicines, etc. are much needed to improve the health status of middle and low income countries, the case of the majority of African countries. So, different solutions can be conveyed by primary healthcare, in order to improve the whole health system.

## Essay

The Alma-Ata Conference popularized primary healthcare (PHC) in 1978. The participants in this Conference affirmed, through a famous Declaration, the values which should characterize this type of care: social justice, right to better health for all, participation and solidarity…[1]. The famous declaration has clearly defined the specificities and characteristics of PHC, the role of the State in promoting this type of care, the position of PHC in a national health system, and the expected objectives by offering this type of local community healthcare. They promoted a health system focused on the individual, with the perspective of the right of everyone to the best state of health that they can achieve, which reinforces equity and solidarity as much as possible and meets the needs of the people [2,3]. Since the declaration of Alma-Ata in 1978, the world has changed considerably. On the one hand, additional difficulties have appeared adding to the complexity (economic recession, climate change, emerging diseases, major epidemics and pandemics…); on the other hand, considerable scientific and technological advances are recorded in all fields. Major technological, organizational and managerial innovations mark the last decades. However, neither the functioning of the health system as a whole nor the interpersonal relationships between practitioners and patients are up to the level of scientific advances, in addition to the innovations and commitments of the international community in favor of the poorest are insufficient [4]. Globalization is straining the social cohesion of many countries, and health systems, key elements of the architecture of contemporary societies, are not working as well as they could and should. It is clear today that, left to their own devices, health systems do not naturally tend to move in the direction of the objectives of health for all, thus deviating more and more from providing care primary healthcare articulated in the Alma-Ata Declaration. Indeed, today´s health systems are developing in directions that do little to contribute to equity and social justice, and fail to derive the best health outcomes from their investments [5,6].

Whether in developed or less developed health systems, we have witnessed since the beginning of the 21^st^ -century gaps that freeze the values of primary healthcare sand hinder their role in a national health system. The WHO report on health in the world of 2008 is another essential reference to take up the primary healthcare concept and the values that go with it, moreover, this report does not fail to highlight the gaps that have arisen in recent decades, and which seriously threaten the philosophy of the primary healthcare system. Although the primary healthcare are healthcare services accessed through preventive mechanisms, we are rather witnessing establishments that provide, more or less, specialized curative care; health systems that set short-term goals and offer fragmented service delivery; questionable governance, which manifests itself in the uncontrolled commercialization of health [7]. Resuming the true values of primary healthcare necessarily involves the realization of aspirations on the ground, among other things, the achievement of the essential objectives of this type of healthcare: social justice, equity, universal access to care, development of the participatory community approach and the promotion of multisectoral actions and intersectoral measures in favor of health. We cannot discuss these dimensions of primary healthcare without limiting ourselves to a recurring obstacle, which resurfaces each time we had the opportunity to carry out reforms at this level of the healthcare offer; this is the question of the modernity of primary healthcare. The modernity of primary healthcare is, from near or far, an objective that often brings together the opinion of the various stakeholders in a national health system: by responding as well as possible to the real health needs of the person seeking care, by improving the performance of the provision of care, as well as by achieving economies of scale for donors.

### Common shortcomings in healthcare delivery; by referring to the Algerian health system example of African health systems

There is an inverse relationship in care; individuals with the most means, whose healthcare needs are often less, consume the most care, while those with the least means and the greatest health problems consume the least. Thus, public spending on health services benefits the rich more than the poor, whether in high-income or low-income countries [8,9]. That said, the neediest are often poorly served! There are explanations for this inverse relationship in healthcare; however, the specifics of the health system tell us more about the causes and consequences of the said inverse relationship. Take the case of the Algerian health system, where there has been the famous principle of free healthcare since the 1970s, which stipulates that all citizens have the same rights to be treated in public healthcare establishments. However, the luckiest are not only the richest, but most often it is the resourceful activating in the informal sector, who opt for irregular actions: favoritism, corruption, etc. to be well served in an area that should not welcome them [4,7]. So the cause of this malaise is plural: the lapse of the law in force, bad governance, bad faith, etc. Wherever social protection is lacking and where users have to pay a large part of care out of pocket, they may find themselves faced with catastrophic expenditure or rather impoverishing care. More than 100 million people fall into poverty each year because they have to pay for their healthcare [10,11]. The specificities and characteristics of the national health system; even tell us more about the hidden face of an inequitable and inefficient social protection system. Taking the case of the Algerian health system, where the taxpayer affiliated with a social security organization is penalized threefold without sometimes receiving what he claims: first when he pays his social security contributions; second, when he pays his taxes; and thirdly, when he pays out of pocket for services purchased in the private sector, without receiving any reimbursement in return [4,7].

As for the citizen who does not benefit from social security, drug expenditure is a burden that swallows up a large part of his purchasing power. We must add an unprecedented phenomenon, which has increased in recent years, which concerns thousands of Algerian patients who go abroad each year for treatment, whether because of the unavailability of treatment or because of likely impacts attributable to neglect or poor quality of care [7,11]. Fragmented care, overspecialization of care providers and narrowness of many disease control programs, etc. discourage a holistic approach to the individual and the family they are caring for and prevent them from assessing the need for continuity of care [12]. Healthcare for poor and marginalized populations is often highly fragmented and grossly underfunded, and development aid often increases this fragmentation [13,14]. The fragmentation of care is a recurring headache for the care seeker, who often improvises by tracing their course of care, to dismantle the organizational obstacles that face their quest for relief, within a system of unorganized healthcare. Although the non-respect of a coordinated care pathway is a real problem for the public care offer, as for the care seeker; it constitutes a plan B to get care as soon as possible, and in the closest healthcare facilities. Taking the case of Algeria, several care seekers do not respect the hierarchy of care; in this case, the first level of the care offered which provides primary care, by presenting themselves directly to hospitals. Taking the case of the Algerian health system, the majority of care seekers do not respect the hierarchy of care, in this case, the first level of the care offered which provides primary care, by presenting themselves directly to hospital emergencies [4,11]. So a narrow fragmentation of care services makes, more or less, organizational accessibility an unattainable objective, and therefore the impossibility of offering continuity of care. This situation is not the result of chance, but rather a shortcoming that characterizes hyper-compartmentalized care systems as well as poorly organized care systems.

A poorly designed system is often unable to ensure safety and hygiene standards usually causing high rates of nosocomial infections, medical errors and other adverse effects, which are an underestimated cause of death and poor health [4]. If health systems in high-income countries are lacking in this regard, then what about the health systems in middle- and low-income countries? Although the situation is different between health facilities in the same region, between regions in the same country and between middle- and low-income countries; the fact remains that, more or less, safety and hygiene standards do are not fully respected. Whether for objective reasons: lack of financial means, lack of specialized human resources, non-application of the law against repeat offenders, etc.; or for subjective reasons: an insufficient culture in the field of health safety and hygiene, a rather weak will to apply the said safety and hygiene standards, a weak involvement of the care recipient and his family, etc. [11,15]. The allocation of resources goes mainly, at great expense, to curative services, neglecting primary prevention and health promotion, yet the latter is likely to allow a reduction of up to 70% in the burden of disease. At the same time, the care sector lacks the necessary skills to mitigate the adverse effects of other sectors on health, and to make the most of the contributions that these same sectors can make to health… we can thus say that we are faced with badly targeted treatments [16]. By analyzing this tendency to go straight towards the curative, we can find explanations sometimes in the philosophy of the health system itself and sometimes in the attitudes and habits of health professionals and the citizen. All the experts agree that acting on the causes of the disease has multiplier effects on public health. So controlling the determinants of health, emphasizing prevention in the broad sense and promoting health are vectors of prevention, which can bring together the interests of all stakeholders in a fairly sensitive sector [4,17].

### Reforms that can further boost the role of PHC

Although the global health systems suffer more and more from the shortcomings that face the supply of care, the fact remains that the countries with intermediate or low incomes, in this case the African countries, suffer more, especially in periods of crises, the latest that of the COVID-19 pandemic [7]. We can underline the different solutions that can be conveyed by the first level of the supply of care, to dismantle the common obstacles that face the supply of health services (Table 1). If the said solutions are thus essential at the first level of the care provision, however, they have multiplier effects on the other levels of specialized and highly specialized care, so they should improve the situation of an entire health system. It should be noted that the degree of development of a health system and its flexibility; are a guarantee of the success of the recommended reforms. For some African countries, the supply of care struggles to guarantee conventional care, so these reforms are seen as utopia”, which requires more means to adopt them. While much has been achieved, according to policymakers, the efforts have revealed a fundamental truth: large-scale interventions and their funding cannot yield better results in the absence of fair and effective health systems [18]. We are thus talking about bridging the gap between aspirations and reality, which requires simultaneous reforms to: respect the values of PHC, respond effectively to the growing expectations of the population in societies in the process of modernization, make the right choices by opting for a multisectoral intervention, and by promoting a participatory approach that excludes no one. We will analyze and discuss these four sets of PHC system reforms, concerning: social values held by the various statements, successful experiences, and guidelines that should improve our dear health.

**Table 1:** the role of primary healthcare reaffirmed in improving health services

Common shortcomings in health services; the obstacles	Fundamental dimensions that make up a primary care system; the solutions
	- Governance of the primary care system; it implies a set of policy implementation characteristics at different levels of the health system, a good interaction between the different components of the health system resulting in good governance of the primary care system.
1. Inverse relationship in care; the neediest are often poorly served!	- Economic conditions for the primary care system; it implies two conditions sine quoi non for access to sufficient and qualitative primary healthcare: the volume of total health expenditure devoted to primary care, as well as the financial conditions of access to care for patients.
	- Equity in health; it refers to the absence of systematic disparities in health between social groups that have different positions in a social hierarchy: racial, social, financial, etc.
2. Depleting Care; financial, geographical and organizational barriers facing the care recipient	- Access to primary care services; they are effective and sustainable solutions to the financial, geographic and organizational barriers that can reduce access to primary care services.
3. Fragmented Care; the case of highly compartmentalized care system	- Continuity of primary care; there are three types of continuity: relational, informational and managerial. Without improvement of continuity, quality universal health coverage will be impossible, in addition it will generate an unjustifiable demand for care in the various primary healthcare services, avoidable hospitalization, overconsumption of drugs, etc.
	- Coordination of primary care; it involves deliberately organizing patient care activities, and sharing information among all those involved in a patient's care, in order to achieve safer and more effective care.
4. Risky care; degree of compliance with health and safety conditions	- Quality of primary care; it depends on the competence, responsibility, initiative and sense of context of each employee in primary healthcare services. It is therefore important to support internal improvement levers. Quality development should be an integral part of all primary care.
	- Primary care workforce development; it includes the profile of professionals providing front-line services and their position in the health system.
5. Poorly targeted healing; curative care is often preferred than preventive care	- Comprehensiveness of primary care services; it emphasizes the real role and the ability of the general practitioner, the pivot of the primary healthcare system, to meet the majority of the current healthcare needs of each patient: he receives patients from his district for diagnosis, dispenses care, advises, guides and develops prevention and health promotion.
	- Efficiency of primary care; An effective primary care system is one that should achieve the maximum of the planned objectives (the level of coverage of care requests) using the minimum of the allocated means: resources, time and money.

Reforms ensure that health systems contribute to equity, social justice and an end to exclusion, primarily by moving towards universal access to care and social security [19]. Universal health coverage is a global ambition that belongs to every assembly that deals with fundamental human rights. Although the said objective of universal health coverage is carried to the highest level by developing countries, however, rich countries are not immune, since ultra-liberal countries show a flagrant deficit in terms of health and social coverage. Taking the example of the world's leading economic power; the United States is one of the only major developed countries that do not have a national health insurance system. About half of Americans have private health coverage through their employer, 7% are individually covered, 14% of citizens over 65 are covered by federal Medicare insurance, and 20% of low-income Americans are covered by Medicaid. However, 9% of the population remains without health insurance to date, i.e. 28 million Americans continue to have no health coverage, and many insurance contracts only offer very minimal coverage [20]. As part of a broad approach to public health, adopted since the Declaration of Alma-Ata in 1978 resulting from the Conference on Primary Healthcare, equitable access to healthcare has become a proclaimed objective, which can improve public health. However, guaranteeing equitable access to PHC should not constitute the miracle solution to stem all the problems of disadvantaged populations. We should not apprehend public health in a fragmented way that is purely medical, since the fragmentation of the problem can lead us towards futile reforms. So the improvement of the state of health of the population requires a global vision, the latter which should seek solutions to the classic problems of social strata with low incomes or in precarious situations: illiteracy, malnutrition, deplorable housing, lack of access to drinking water, insufficient sanitation services... Therefore, improving public health requires equitable access to PHC, at the same time improving the socio-economic conditions of citizens [21].

Service delivery reforms are those that reorganize health services in the form of primary healthcare, i.e. around the needs and expectations of the population, to make them more socially relevant and more responsive to changes in the world, while producing better results. Several events and variables can, more or less, change the efficiency of an entire health system. We have always witnessed the emergence of communicable diseases, which spread locally, nationally or internationally (endemic, epidemic or pandemic), and all these phenomena are serious tests for judging the effectiveness of the health system. The effect of surprise when faced with an unprecedented pathogen; is an undesirable situation for sector managers, which could weaken the efficiency of an entire health system. By addressing the case of the COVID-19 pandemic, all these undesirable factors come together to render health systems, even the most developed ones, powerless in the face of such an unprecedented phenomenon. The result is that the health system should continue to operate, however with an efficiency deemed insufficient, such as the bitter observation of the various health systems of the countries of the world: more than 452 million cases recorded and more than 6 million deaths for this first half of March 2022 [22,23]. We also point out that societies are in permanent motion, however, the plural transitions which cyclically shook a society a few centuries ago, have become almost permanent since the second half of the 20^th^ century, and this is because of several variables which push this movement towards metamorphosis, or rather towards chaos… [4,7]. So offering care for citizens is no longer enough, the most important thing is to anticipate the incidence of the disease, by acting on the determinants of the disease. In the same vein, PHCs offer several opportunities: early detection of serious pathologies (cardiovascular diseases, different types of cancer, chronic diseases); the detection of people at high risk of developing cancer or another non-communicable disease. This is the true mission of a modern and effective PHC system, which could save thousands of people, and this by acting on the causes of the disease by anticipating its incidence and, by avoiding as much as possible curative care and late complications. Thus, primary healthcare is increasingly seen as an intelligent way to put health development back on track, by best meeting the expectations of the population [21].

Public policy reforms that make communities healthier, combining public health actions with primary healthcare, and pursuing healthy public policies in all sectors are badly needed. The United Nations Millennium Declaration reconnected with the values of equality and social justice, setting an ambitious goal: to help each other to eradicate poverty at the global level. A declaration which set eight very ambitious objectives to achieve the said main objective “eradicate extreme poverty and hunger”, and this by emphasizing health as a sine qua non-condition for reducing poverty [24,25]. So the eight Millennium Development Goals/MDGs are interrelated; all affect health, and conversely, health affects all the MDGs. Thus a healthy citizen who lives in a healthy environment could contribute effectively to the economic development of the country, and vice versa, the economic development of the country could benefit all citizens by gradually eradicating poverty and hunger. To date, the MDGs have significantly improved people's quality of life: a reduction in global poverty by half five years before the 2015 deadline; approximately 90% of children living in developing regions now benefit from a primary education; a marked improvement in all health indicators; a reduction in the likelihood of a child dying before their fifth birthday by 50, which means that approximately 17,000 children are saved every day [24,26]. Such multisectoral efforts, supported by international involvement, should better target public policies towards the achievement of sustainable development goals (economic, social and health, etc.) that benefit all. So we can say that we have succeeded in the transition from the Millennium Development Goals/ MDGs to the Sustainable Development Goals / SDGs.

Leadership reforms are the reforms that replace both the disproportionate pursuit of short-term results on the one hand and the disengagement of public authorities on the other, by introducing inclusive, participatory, negotiating leadership. Although the problems that weaken health systems are more or less different from high-income to middle-income and low-income countries, all health systems face temporary, recurrent or structural obstacles. Since the end of the 20th century, the countries of the northern hemisphere have been faced with the phenomenon of ageing; the natural result of the baby boom after the Second World War, an almost continuous decline in the birth rate, a continues gains in life expectancy since the end of the Second World War, etc. However, the combination of these and other factors should make those who manage the health sector more responsible, to offer seniors better care by updating the map of diseases, planning health priorities, effectively managing the social security funds, organizing the best financial means and human resources, etc. to cope with the long-term treatments they require in this age group, which further weaken the already fragile health systems, leading to increased health expenditure [27]. The countries of the southern hemisphere have other concerns, or rather quasi-permanent financial and other organizational problems. Regarding health financing, the majority of these countries often face insufficient funding for health and financial barriers that exclude many poor people from accessing health services. At the level of the organization of the health system, the question that comes up time and time again is: how to organize health services to provide equitable and effective healthcare? [7,28]. Beyond these classic problems, sector leaders should look to solutions that can save millions of lives around the world: preventing chronic diseases, by focusing efforts on the social and environmental determinants of health. Achieving this objective requires multisectoral collaboration, decentralized decision-making, a solid information system and a participatory approach that does not exclude anyone from a current debate [2,7].

### Conclusion

Within the framework of this article, we have confined ourselves to seeking the answers on how to resume the ambitious project of primary healthcare/PHC, decreed for the first time in the famous Declaration of Alma-Ata in 1978, but this time in another context, characterized by the emergence of an unprecedented pandemic, record poverty rates, health systems weakened by growing demand and limited resources, etc. Today, primary healthcare is increasingly seen as an intelligent way to put health development back on track, however, several constraints face an almost continuous reform of the health system, in this case, that of the PHC system: health expenditure has increased, generally, faster than GDP in recent decades, helped by the revolution in the field of information and communication technologies/ICT, the requirements of the care recipient for a better quality of care, the growing threat of emerging diseases and epidemics, the repercussions of trade agreements on the availability and the prices of food and pharmaceutical products... Therefore, we can no longer act unilaterally, multisectoral efforts at the national level should be encouraged, to launch the debate on the multiple causes of disease, which determine the health status of the population, these determinants of health which require multidisciplinary studies to understand their influences, and thus plan corrective measures to improve public health. The international agreements must be on: the environment, prices of basic foods and medicines, coordination of efforts to develop medicines and vaccines, humanitarian aid to refugees, etc. are welcome, which should improve the state of the health of the countries which are struggling to cover the health needs of their inhabitants, in this case the African countries, as well as the living environment of all humanity.
